# Lived experience and attitudes of people with plantar heel pain: a qualitative exploration

**DOI:** 10.1186/s13047-020-0377-3

**Published:** 2020-03-06

**Authors:** Matthew Cotchett, Michael Skovdal Rathleff, Matthew Dilnot, Karl B. Landorf, Dylan Morrissey, Christian Barton

**Affiliations:** 1 Discipline of Podiatry, School of Allied Health, Human Services and Sport, La Trobe University, Melbourne, 3086 Australia; 2 Center for General Practice, Department of Clinical Medicine, Aalborg University, 9000 Aalborg, Denmark; 3 SMI, Department of Health Science and Technology, Faculty of Medicine, Aalborg University, Aalborg, Denmark; 4 La Trobe Sports and Exercise Medicine Research Centre, School of Allied Health, Human Services and Sport, La Trobe University, Melbourne, 3086 Australia; 5 Sports and Exercise Medicine, QMUL, London, E1 4DG UK; 6 Department of Surgery, St Vincent’s Hospital, University of Melbourne, Melbourne, Australia

**Keywords:** Plantar heel pain, Plantar fasciitis, Qualitative research, Interview, Patient education

## Abstract

**Background:**

Plantar heel pain is a common source of pain and disability. Evidence-based treatment decisions for people with plantar heel pain should be guided by the best available evidence, expert clinical reasoning, and consider the needs of the patient. Education is a key component of care for any patient and needs to be tailored to the patient and their condition. However, no previous work has identified, far less evaluated, the approaches and content required for optimal education for people with plantar heel pain. The aim of this study was to gather the patients’ perspective regarding their lived experience, attitudes and educational needs in order to inform the content and provision of meaningful education delivery approaches.

**Methods:**

Using a qualitative descriptive design, semi-structured interviews were conducted with participants with a clinical diagnosis of plantar heel pain. A topic guide was utilised that focused on the experience of living with plantar heel pain and attitudes regarding treatment and educational needs. Interviews were audio recorded, transcribed verbatim and analysed using the Framework approach. Each transcription, and the initial findings, were reported back to participants to invite respondent validation.

**Results:**

Eighteen people with plantar heel pain were interviewed. Descriptive analysis revealed eight themes including perceptions of plantar heel pain, impact on self, dealing with plantar heel pain, source of information, patient needs, patient unmet needs, advice to others and interest in online education. Participants revealed doubt about the cause, treatment and prognosis of plantar heel pain. They also expressed a desire to have their pain eliminated and education individually tailored to their condition and needs. Respondent validation revealed that the transcripts were accurate, and participants were able to recognise their own experiences in the synthesised themes.

**Conclusion:**

Plantar heel pain has a negative impact on health-related quality of life. Participants wanted their pain eliminated and reported that their expectations and needs were frequently unmet. Health professionals have an important role to be responsive to the needs of the patient to improve their knowledge and influence pain and behaviour. Our study informs the content needed to help educate people with plantar heel pain.

## Background

Plantar heel pain (PHP) is common in the community with an estimated prevalence between 3.6 and 9.6% [1–4], although there is a paucity of high-quality prevalence studies. PHP has a negative impact on health-related quality of life and is frequently associated with significant disability due to pain, and psychological distress [4–7]. The condition can be long-standing [8] and one study found that 1 in 5 patients took several days of sick leave due to their heel pain [9]. Current evidence-based treatments for PHP, supported by systematic reviews, include foot orthoses [10], foot taping [11], and extra-corporeal shockwave therapy [11, 12]. However, a recent network meta-analysis showed that effect sizes are small and there is no clear superiority of any available treatment [13].

Evidence-based treatment decisions for people with PHP should be guided by the best available evidence, expert clinical reasoning, and take into account the needs of the patient [14]. Health care that is responsive to the values, preferences and needs of the patient improves patient experiences [15–18]. Education is a key component of care for any patient and needs to be tailored to the patient and their condition. However no previous work has identified, far less evaluated, the approaches and content required for optimal education for people with PHP.

As an initial step towards developing optimal care, there is a need to understand the lived experience and attitudes of people with PHP. The aim of this study was to explore the lived experience and attitudes of people with PHP in order to inform the provision of meaningful education delivery approaches and resource formulation.

## Methods

### Ethics

The Human Ethics Committee at La Trobe University approved the study (S17–075) and all participants signed informed consent.

### Study design

This study was a qualitative descriptive design with semi-structured interviews and followed the Consolidated Criteria for Reporting Qualitative Studies reporting guidelines [19].

### Sampling and recruitment

Participants in the greater metropolitan Melbourne and the central regional area of the State of Victoria, Australia were recruited using advertisements in community centres, primary care clinics and La Trobe University. Interested individuals called or emailed the lead author and an appointment was made to screen for eligibility. The Participant Information Statement and topic guide (Table 1) was provided to participants 1 week prior to the screening process. No relationship was established with the participant prior to the screening process and data collection. No participants that met the eligibility criteria refused to participate.

**Table 1:** Topic guide to facilitate the semi-structured interview

1. Can you tell me about the problems you have been having with your heel?	
2. What do you think caused your heel pain?	
3. What do you think is happening in your heel?	
4. Has your heel pain changed things for you in your life?	
5. What do you do for your heel pain?	
6. What do you understand about your treatment options – which might or might not be effective and why?	
7. What or who has influenced your treatment decisions (clinician, internet, family, etc)?	
8. What advice would you give to someone else who has similar issues with their heel?	
9. Do you regret any treatment decisions (or happy with your decisions)?	
10. What do you want from a clinician for whom you may or may not have consulted for your PHP?	
11. Who has provided your education and what specific education resources have been provided to you?	
12. Have you used the internet to find additional information and if so where did you go and what did you find?	
13. What do you wish you had learnt sooner about your heel pain?	
14. How do you think education provided about your condition can be improved?	
15. If a website was to be built, which provided education about your condition	
• What information should be included?	
• How would you like the information to be presented?	

A purposive sampling frame was utilised to capture a wide range of viewpoints that was representative of the population based on sex, age, duration of symptoms, and previous treatment. This was used to guide recruitment of participants with a clinical diagnosis of PHP [20], including: (i) aged 18 years and above, (ii) symptoms greater than 1 month duration, (iii) a score of > 20 mm on a Visual Analogue Scale for average pain over the past 7 days, and (iv) pain on palpation of the medial tubercle of the calcaneus. Exclusion criteria included PHP that might be systemic in origin, and/or (ii) related to more sinister causes of PHP such as tumours, infections and fractures.

### Data collection

Individuals who met the criteria, and provided informed consent were subsequently interviewed. Prior to interview, participants completed a Visual Analogue Scale for pain and the Foot Health Status Questionnaire for foot pain and foot function [21]. The topic guide (Table 1) was developed via a literature search of studies that evaluated the experience of people with foot osteoarthritis [22], the educational needs of people following knee and hip replacement [23] and discussion amongst the research team. In addition, the topic guide was guided by the clinical and academic experience of the research team.

Interviews were semi-structured with open-ended questions. Data were collected until no new themes materialised. All interviews were conducted by MC and lasted between 6 and 31 minutes (mean = 15). No field notes were gathered during the interview and no repeat interviews were conducted.

### Data analysis

The interviews were audio recorded, transcribed verbatim and analysed using a Framework approach [24, 25]. First, the lead author (MC, a registered podiatrist with 20 years of experience) checked all transcripts for errors by listening to the audio files and reading the transcript. To become familiar with the dataset, MC re-read the transcripts and made notes to record impressions. Second, a thematic framework was established by assigning a ‘code’ to a key issue that captured the essence of that theme. Themes were derived from the data rather than identified in advance. Third, ‘indexing’ was conducted, which involved grouping together codes that were conceptually related and assigning the cluster an overarching theme. A second author (CB, a registered physiotherapist with 14 years of clinical experience and previous experience conducting qualitative research) independently reviewed the codes and indexing, against the manuscripts, to verify their development. Fourth, a spreadsheet was constructed and the qualitative data was ‘charted’ into a matrix (Supplementary File 1) by summarising data by theme from each transcript [25]. Quotes were extracted from the transcripts to highlight the participant’s voice and contribute to credibility and transparency of the research. Fifth, the qualitative data were ‘interpreted’ by MC and CB to explore interesting ideas and map connections between themes [25]. Respondent validation [26] was conducted by providing all participants with a copy of their transcript and a spreadsheet that included emerging themes.

## Results

Eighteen participants were recruited between May 25th, 2017 and July 20th, 2018. Baseline characteristics are listed in Table 2. Participants had a mean ± SD age of 58.2 ± 6.6 years and 66% were female. Thirty-nine percent of participants had a history of hypertension, while 5% had a history of heart disease, hypercholesterolaemia, and thyroid disease and 11% had a history of gout. No participants with infections, fractures or tumours were identified based on the subjective and objective evaluation. Member checking highlighted that the transcripts were accurate, and participants were able to recognise their own experiences in the synthesised themes.

**Table 2:** Characteristics of participants

Variable	*N* = 18
Age	58.2 (6.6)
Gender, n (%) female	12 (66%)
Height	169.3 (9.4)
Weight	79.5 (21.3)
BMI	27.8 (5.7)
Duration of heel pain (months)	15.9 (16.3)
Education (years)	16.1 (3.6)
First step pain, 100 mm VAS^a^	45.6 (34.0)
Average pain today, 100 mm VAS	34.3 (25.4)
Average pain past 7 days, 100 mm VAS	43.9 (24.7)
Foot Pain, FHSQ^b^ (100 point scale)	51.4 (17.5)
Foot Function, FHSQ (100 point scale)	52.8 (30.4)

Note: values represent mean ± SD, unless otherwise stated

*Abbreviations*: *FHSQ* Foot Health Status Questionnaire, *VAS* Visual Analogue Scale

^a^Higher values indicate greater severity of pain

^b^0 corresponds to the worst foot health, 100, the best

The Framework analysis resulted in eight themes with 43 sub-themes (Supplementary file 1). The eight themes included ‘perceptions of PHP’, ‘impact on self’, ‘dealing with PHP’, ‘source of information’, ‘patient needs’, ‘patient unmet needs’, ‘advice to others’ and ‘interest in online education’.

### Theme 1. Impact of PHP

Participants described first step pain as the most common symptom. The experience of PHP had a negative impact on health-related quality of life, especially on physical function including a reduced ability to walk, run, stand for long periods, go on holidays and entertain friends:

*“I don’t feel as strong in my whole body. I have a bit more trouble lifting, trouble walking, especially down slopes or downstairs, not so bad going up stairs or up slopes, but activity has certainly been slowed right down” (Participant 17).*

A reduction in physical activity was associated with negative emotions and feelings including sadness, hopelessness and frustration:

*“I’m a mouse on a wheel. I can’t seem to get off. I don’t know what to do. I don’t know how to lose weight without moving, and how do you move without the pain? So yeah, sometimes I’m a bit exasperated by it” (Participant 15).*

The experience of pain was also an obstacle to being socially active, with some participants expressing a feeling of social isolation.

### Theme 2. Perceptions about PHP

Several subthemes emerged from the participants’ perceptions of their PHP including beliefs about the cause, the underlying pathology and meaning of pain. Numerous causes were proposed by participants including being overweight, a change in the level of activity, standing for long periods, walking on hard, soft and uneven surfaces, and walking barefoot:

*“Probably I was overdoing it. I increased my walk because I used to do around the three to four kilometres, pushing every now and then. But since I retired, I’ve been going five and at times eight (kilometres), and then walking on both hard surface and the sand” (Participant 4).*

*“I just thought it was because I put on a lot of weight during that time” (Participant 14).*

Similarly, participants reported numerous descriptions of the underlying pathology including plantar fasciitis, bruised heel, broken bone, heel spur and nerve irritation but many were unsure:

*“Well, I really don’t know. I didn’t understand what it was. I knew it had something to do with the plantar fascia” (Participant 11).*

Participants were also asked about the meaning of their pain with some highlighting that pain was a signal of a threat or warning sign.

*“I feel like pain is my trigger to just try and do something a little bit different” (Participant 18).*

### Theme 3. Coping with PHP

Participants described a myriad of interventions for PHP including orthomechanical, physical therapies, exercise, pharmacological and strategies to modify their activity. The most common interventions were foot orthoses, exercise, taping and advice regarding footwear, although a few participants combined multiple interventions. Participants’ descriptions of the exercise prescription, which included either stretching or strengthening, were variable. Some participants reported that exercise provided short-term relief, while one participant questioned the effectiveness of exercise:

*“Either I’m not particularly disciplined at doing that (stretching and strengthening) or it just didn’t actually help – maybe I was looking for a quick fix and it didn’t happen quickly enough, and I became a bit frustrated with that” (Participant 18).*

Foot taping was reported to be associated with short term pain relief, although participants reported adverse events such as skin irritation. Prefabricated foot orthoses had been explored by participants but with mixed success:

*“Well, talk about painful, they were dreadful and then most of my shoes, I couldn’t wear because my instep is too high, and I couldn’t get the foot in the shoe with the orthotics in it” (Participant 5).*

*“The thing I’ve had the most success with is some orthotic inner soles that are very rigid and seem to hold my foot still. That seems to give me the most support” (Participant 18).*

Footwear played a key role in alleviating pain with participants highlighting the importance of wearing supportive shoes with a small degree of heel elevation, and caution was expressed when walking in flat unsupportive shoes:

*“Go invest in a good pair of shoes firstly. Never walk barefoot or in thongs and even if you get up in the middle of the night, make sure you put a shoe on to walk around” (Participant 7).*

Tension existed in participants’ responses relating to the role of modifying activity and or rest. While some participants modified their behaviour by limiting or eliminating an activity, others questioned the importance of rest:

*“I didn’t realise that this would help but I’ve started running and I’ve stopped eating sugar and I don’t think the sugar has a relationship but what it has done is help me lose weight and get healthy” (Participant 17).*

*“Maybe I just didn’t give it long enough, but I did have a week of total rest and it didn’t help my foot and my brain nearly went into a massive meltdown” (Participant 18).*

Overall, a common theme reported by participants was the implementation of a simple trial and error approach. This was associated with a sense of frustration about the lack of consistency in treatment approach and the absence of treatments known to work for patients:

*“But honestly, if someone told me to mix up a special drink ‘cause that’s what was gonna fix it, that’s probably what I’d do as well. So, I probably am trying anything. It’s a bit like spin the wheel and try your luck. I’ll try it all” (Participant 18).*

### Theme 4. Source of information

Most participants identified allied health and medical professionals as their main source of information, although some participants perceived not being taken seriously:

*“I did actually go to the doctor once and described it to him, but – yeah, he just said massage (the foot). The GP didn’t look in the slightest bit interested, really” (Participant 6).*

Participants frequently used the internet, although information was confusing, conflicting and rarely useful:

*“A lot of descriptions of very long words that I didn’t really understand … it gave a description about what it was, but actually I didn’t find anything that was really telling me what to do” (Participant 17).*

Discussing and sharing the experience of having PHP with other people who have experienced PHP provided opportunities to exchange practical advice and harness support:

*“The idea is to gather as much information as possible from various sources regardless of whether they are a podiatrist, doctor, friend, or family, because sometimes you may get the best advice from people who have experienced this sort of problem” (Participant 4).*

### Theme 5. Patient needs

Participants provided rich descriptions of their treatment needs with three sub-themes emerging. First, participants wanted clear explanations of the diagnosis, aetiology, prognosis and treatment options:

*“If I had a better idea, better understanding of what was actually happening with it, I think that would have guided me a lot better in what I was doing about it” (Participant 3).*

*“I know that’s a very subjective sort of thing, but to have some idea of – okay, this could take months, it could take three months, it could take six months – just to have some sort of idea, I think, of how long it might take ‘cause I really thought I’d be over it by now” (Participant 10).*

Second, people with PHP wanted face-to-face consultations with a health professional that appeared interested, asked appropriately directed questions, and delivered information in a clear, confident and easy to understand manner. Online resources were more cautiously viewed, with material being seen on a spectrum from valuable to requiring caution:

*“Not everything that you read on the internet is possibly correct, so I just dealt with it. I mean, some physicians are not always correct too, but I just felt more secure in actually seeing someone and talking to them face to face rather than reading it off the internet” (Participant 13).*

Some participants expressed a desire to be given educational resources, such as patient handouts, which provide an outline of the plan recommended by the clinician that can be revisited when suitable for them:

*“I want some clear information. Maybe I have seen in some instances for other situations where people have a handout and there are a couple of little photos with brief explanations and maybe the five top suggestions, something to take home” (Participant 5).*

Third, while participants wanted clear education-based information for their PHP, they fundamentally want to have their pain eliminated:

*“I mean, there may be no magic bullet here to say we can get rid of it, but if there’s a way to stop the pain, so you can be more active and – yeah, I’m all ears. I’m open to whatever is available (Participant 1)”.*

### Theme 6. Patient unmet needs

Participants described unmet expectations regarding explanations of the underlying pathology, causal factors, and descriptions of interventions:

*“A lot of the education is being well, “Do this or do that,” but without really explaining what it is that you’re doing and why you’re doing it and what you’re supposed to achieve” (Participant 3).*

*“Here’s half a dozen things that you should try in order of how frequently they assist people with similar problems,” I think. So, like a bit of a format or a plan of what to do and when to move to the next step” (Participant 5).*

Participants expressed frustration with conflicting information regarding the best approach to manage PHP with a spectrum of messages being delivered by clinicians and resources available online:

*“I mean there’s so much conflicting information on the internet, trying to put that into context with what doctors told me, what my physio friend at work told me and reading different things on the internet, trying to relate that to my condition, and work it all out” (Participant 3).*

Some participants wanted access to clear and simple education-based resources as current resources online were not considered trustworthy or written from an Australian perspective.

Participants’ understanding of the evidence base could be considered poor. They had limited knowledge of what treatments are deemed to be effective although a subtheme emerged that corticosteroids, foot orthoses, exercise and surgery might be effective.

### Theme 7. Advice to others

A strong theme emerged regarding the importance of seeking early diagnosis and advice from a health professional:

*“Get a good diagnosis and someone who knows what they’re doing. I suppose looking back, getting a firmer diagnosis at the beginning maybe, trying to get some information that seemed to relate more specifically to your own condition because I found that I was never really quite clear on what advice I was getting really related to what I had because of confusion over what it was” (Participant 3).*

Other subthemes emerged including the importance of wearing supportive shoes and avoiding walking barefoot. There was tension in participants’ responses regarding the use of foot orthoses with both positive and negative experiences. Similarly, some participants advised others to avoid exercise, while some suggested to continue with a certain level of exercise.

### Theme 8. Interest in online education

Participants were asked about online education for people with PHP. Most participants had sourced information from health professionals and wanted face-to-face consultations (Theme 5) but a theme emerged that this was often supplemented with online education.

Consistent with the needs of participants seeking advice and support from health professionals, participants interested in online education wanted a clear definition of PHP including the characteristic features of the condition, (e.g. diagnostic criteria, typical location of symptoms and casual factors) so that they could equate their presentation to that of a known diagnosis.

*“I want videos that show where pain is on the foot, having that pointed out – drawn on a foot, so I can replicate it on mine, all that sort of – if your pain is here, it could be this. If it’s here, it’s probably not this” (Participant 18).*

Participants also wanted to learn more about treatments including where to go for professional advice and what treatments are effective at different stages:

*“I want to see like research-based information. I did find my physio therapist was good at saying, “Oh, this is the latest evidence-based practise for how to help with your hamstring tear.” We didn’t really go to heel descriptions of research. So, I think it should be informed by that and that that should be a clear link” (Participant 5).*

Some participants discussed the importance of providing more detailed information to explore issues at a deeper level:

*“Pretty much all of the websites that I looked at had the same sort of material just in different words, different diagrams, but not really getting down any deeper than that, and I think I really wanted to understand a bit more about it (Participant 3)”.*

Table 3 is a summary box of the key components of an educational intervention for people with PHP interested in online education based on the interviews.

**Table 3:** Components of an educational intervention

Checklist	
The content	
Provide a definition of plantar heel pain, including classic features that differentiate it from other conditions	
Include information about what is effective and what is ineffective	
Provide treatment options at different stages of the condition	
Define the role of various health professionals that can provide advice	
Provide access to information that allows greater knowledge exploration	
Include stories of people who have lived with PHP	
Include videos that demonstrate exercises (e.g. stretching)	
The features	
Use simple, clear, and easy to understand terminology	
Concise and easy to read	
Include good use of illustrations	
ONLINE elements	
Perform a walkthrough of the website	
Include good use of written, audio and visual illustrations	
Ensure there is a clear statement of quality assurance	
Regular updates	
Provide handouts that can be downloaded for easy reference to key issues	

## Discussion

This is the first qualitative study to explore the experiences and attitudes of people with PHP. Participants’ responses revealed that although PHP negatively impacted their health-related quality of life, including a range of disruptions to the physical, mental and social aspects of their lives, they had doubt about the cause and nature of PHP. A myriad of interventions were trialled by participants with foot orthoses, taping, and exercise being prominent. Participants sourced information largely from health professionals but had variable experiences of how useful it was and how well it was provided. This was often supplemented with online material, which was considered confusing and rarely useful. Participants also expressed the desire to have their pain eliminated and education delivered that was clearly communicated and individually tailored to their condition and needs.

Our results indicate uncertainty among people with PHP regarding their diagnosis, as well as its cause, prognosis and treatment options. This resulted in participants reporting that they experienced frustration and they struggled to find an appropriate treatment plan to meet their needs. This is not surprising given the lack of clarity about the underlying pathology [27], limited effectiveness of available interventions in the literature [13, 20, 28], factors associated with PHP [29], and prognosis [8, 20, 30]. Varied beliefs, uncertainty, coupled with a desire for better understanding, highlights the need to rethink how PHP is viewed, assessed and treated.

Our findings are consistent with other groups of people who have chronic disease, who express the need for information to understand what was wrong, have a realistic idea of their prognosis, learn how to prevent their condition, better understand the outcomes of various treatments, and identify who to see and sources of help [31]. Our findings are also consistent with the lived experience of people with rheumatoid arthritis where education is recognised as being essential to maintain optimal foot health, although the needs of the patient are not always being met [32]. A lack of appropriate education about specific foot related interventions was also associated with fear and anxiety in people with rheumatoid arthritis [32].

The results of our study have immediate implications for clinical practice, with participants providing rich descriptions of their clinical needs. Although, for many of the participants interviewed, which were largely representative of the PHP population, their needs were largely unmet. However, it is unclear if this unmet need related to the choice and combination of treatments implemented, the manner in which the participant was educated, or an inability or readiness of the participant to engage in their treatment and work towards health behaviour change [33, 34].

To help meet patient needs, it is essential that evidence based clinical practice guidelines be established for PHP that include the best available evidence from high quality trials synthesised with expert opinion and the ‘patient voice’. If health professionals are guided by clinical practice guidelines it might keep treatments within a narrow range of practice and the process of care may become more consistent across different health professionals. In addition, health professionals need to communicate evidence-based information in a clear, and understandable manner that is responsive to their needs.

The impact of PHP extended beyond physical limitations typically targeted by interventions in clinical trials [11, 13], negatively impacting on emotions, feelings and thoughts. Psychological factors have been found to be associated with increased pain and reduced foot function in people with PHP [6, 7]. Additionally, symptoms of depression and anxiety are associated with a poorer prognosis for a range of musculoskeletal conditions, which underscores the potential importance of such factors in the patient’s experience of pain [35]. Accordingly, health professionals should view the patient through a broader biopsychosocial model and attempt to ascertain the dominant contributors of this model to the patient’s presentation [36].

Our study informs methods to educate people with PHP using different media. Future research may consider the provision of written, audio and visual resources including a freely accessible online resource, which is reported to be effective for other conditions. Printed or electronic information can improve knowledge and understanding [18], confidence and coping ability [18], reduce anxiety [37], improve social support, health behaviours, adherence to treatment recommendations and clinical outcomes [38]. In people with diabetes, brief foot care education has been shown to positively influence patient knowledge and self-reported patient behaviour [39]. In addition, written education, co-designed with patients, has been found to be effective for improving foot health in people with Type II diabetes, while interactive education was found to improve understanding of important preventative measures in this population [40]. Similar education approaches may be of benefit to people with PHP.

Our study needs to be viewed in light of some limitations. First, the predominantly negative experiences of the sample might have reflected the recruitment of symptomatic participants, disgruntled with their experience, who were seeking guidance, although this group is especially important to improve treatment for. Second, the closed-ended nature of some questions might have limited deeper responses from participants, which might explain the brevity of some interviews. However, we used a qualitative descriptive research design where the goal was to obtain straightforward and minimally theorised answers to questions relating to the who, what and where of the participant’s experience [41]. This is in contrast to other qualitative research designs that use a more interpretative approach to explain phenomena (e.g. phenomenology, grounded theory, or ethnography) [41].

## Conclusion

The data revealed that PHP negatively impacts health-related quality of life. Participants reported that they wanted their pain eliminated, although their expectations and needs were frequently unmet. Our findings point to the potential importance of providing patient-centred care that considers, and is responsive to, patients’ needs and expectations as well as giving a clear guidance about delivery and content.

## Supplementary information

**Additional file 1.** The Framework Matrix - summary of themes and subthemes.

## Data Availability

The datasets used and/or analysed during the current study are available from the corresponding author on reasonable request.
