# Supplementary material for: Lived experience and attitudes of people with plantar heel pain: a qualitative exploration
Source: J Foot Ankle Res. 2020 Mar 6;13:12. doi: 10.1186/s13047-020-0377-3 (PMC7059663; doi:10.1186/s13047-020-0377-3)
Supplement: Supplementary file 1 — Additional file 1. The Framework Matrix - summary of themes and subthemes. [file 13047_2020_377_MOESM1_ESM.docx]

**Theme 1: Impact on self**

| **Participant** | **SUBTHEME (CODE)** | | | | |
| --- | --- | --- | --- | --- | --- |
|  | **Symptoms** | **Physical** | **Mental** | **Social** | **Other** |
| 1 | Severe pain; stabbing pain ; knife like | Weight gain; reduce function; | Loss of pleasurable actvities; dejected; sense of helplessness; frustrated | Reduced social interaction with partner |  |
| 2 |  | Reduced function; limits walking; |  | Isolation *"So I don’t go out very much because of the pain"* **Q** |  |
| 3 | First step pain; pain increases with exercise | Reduced walking; difficulty walking; loss of condition; and reduced function *"I think my overall health has been impacted because I’m just not doing enough movement at all and because of my sitting for job, I think that’s made that even worse*" **Q** |  |  |  |
| 4 | First step pain | Limited walking; | Loss of pleasurable activities such as travelling |  |  |
| 5 | Constant pain;; excruciating pain |  | Loss of pleasurable activities such as running |  |  |
| 6 |  | Limited exercise; |  |  |  |
| 7 | First step pain which lessens with initial exercise |  |  |  |  |
| 8 |  | Limited walking and exercise; causes a limp; | Irritated |  |  |
| 9 | First step pain | Limited exercise; | Disappointed; reduced happiness; sadness |  |  |
| 10 |  | Limits walking | Debilitating |  |  |
| 11 | First step pain | Limits walking; | Loss of pleasurable activities such as standing to cook |  |  |
| 12 | An ache and a stabbing pain; noticeable when getting out of bed; constant pain | Limits walking; reduced function | Constant pain is annoying; impact on ability to concentrate |  |  |
| 13 | First step pain; pain eases with initial exercise |  |  |  |  |
| 14 | First step pain; shooting pain | Reduced function; walking limited | Catastrophising *"I'm going to be in pain forever"* **Q** |  | lack of insurance limited access to treatments |
| 15 | First step pain; heel pain; pain eases with initial exercise | Reduced function; reduced physical exercise; weight gain | Deflated; Weight gain has been depressing; Frustration: *"I'm a mouse on a wheel. I can’t seem to get off. I don’t know what to do. I don’t know how to lose weight without moving, and how do you move without the pain? So yeah, sometimes I'm a bit exasperated by it"*. **QQ** |  |  |
| 16 | First step pain | Reduced function; limits walking *"I tailor my activities around my heels"*. **QQQ** | Loss of pleasurable activities; cranky |  |  |
| 17 | Pain first thing in the morning; pain eases with initial exercise | Reduced strength and reduced function *"I don’t feel as strong in my whole body. I have a bit more trouble lifting, trouble walking, especially down slopes or down stairs, not so bad going up stairs or up slopes, but activity has certainly been slowed right down*" **QQ** | Low mood at times; |  |  |
| 18 | Pain first thing in the morning; | Limits exercise *"I’ve spent the last few months trying to find other forms of exercise that I can do that don’t aggravate my heel in the same way*" **Q**; limits walking; | Loss of pleasurable activities; low mood at times *"my partner would say he thought I was depressed from it"* **Q**; cognitions *"I feel like I have to rethink everything"* **Q** | Negative impact on social network*"I really miss the people and the friendships that I had there"* ***Q*** | Restricted to certain shoes "So, it means I can’t wear all of my shoes. I have a lovely array of shoes and I’m wearing about three pairs on high rotation" **Q** |

**Theme 2: Perceptions about PHP**

|  | **Subtheme** | | | |
| --- | --- | --- | --- | --- |
| **Participant** | **Perceptions of the cause** | **Perceptions of the nature of the pathology** | **Perceptions of pain, exercise and tissue damage** | **Other** |
| 1 | age related; standing on feet all day; running at a certain pace; running on uneven surfaces | Spur; ripped plantar fascia; broken bone |  |  |
| 2 | No idea; | No idea |  |  |
| 3 | Associated with a period of overuse | Plantar fasciitis; inflammation |  |  |
| 4 | Overuse from walking; walking on hard surfaces; walking on soft surfaces "*Probably I was overdoing it. I increased my walk because I used to do around the three to four kilometres, pushing every now and then. But since I retired, I’ve been going five and at times eight (kilometres), and then walking on both hard surface and the sand" Q* | Inflammation of the plantar fascia; plantar fasciitis |  |  |
| 5 | related to a previous hamstring injury; reduced calf strength and tightness; | Plantar fasciitis; nerve dysfunction; |  |  |
| 6 | Weight gain | Tendon or ligament tension |  |  |
| 7 | Uncertain; walking barefoot; shoes without arch support | Inflammation of a tendon |  |  |
| 8 | Related to oversuse of the heel; | Uncertain; Heel spur; overstretched tendon; inflammation | |  |
| 9 |  | Bruised heel | Pain demands attention to re-evaluate the situation |  |
| 10 | Heel irritated by new generic orthoses | Plantar fasciitis; fat pad irritation; bruising of the heel | Pain not associated with tissue damage; exercise provides happiness |  |
| 11 | No idea; *Well, I really don’t know. I didn’t understand what it was. I knew it had something to do with the plantar fascia* | Plantar fasciitis |  |  |
| 12 | Reduced capacity to withstand the load following another injury; reduced calf strength following injury; compensation for previous injury; related to walking barefoot or using Vibran five fingers | Uncertain; inflamed plantar fascia; imaging findings are linked to symptoms | Pain is associated with tissue damage | Imaging findings provide confidence in the diagnosis; imaging findings are linked to symptoms |
| 13 | Over exercising; wearing hard soled shoes | Uncertain; bone issue; muscular issue? |  |  |
| 14 | Uncertain; weight gain. "*I just thought it was because I put on a lot of weight during that time"* | Bone spur or growth on the heel |  |  |
| 15 | Wearing flat shoes; pain due to tightness in the calf; high arched feet | Inflammation of the plantar fascia | Pain not associated with tissue damage |  |
| 16 | Old age; peripheral vascular disease; arterial narrowing; |  |  |  |
| 17 | Being overweight and standing on feet all day | Plantar fasciitis; spur developing on the heel; |  |  |
| 18 | High impact exercise; period of overuse; walking barefoot over summer; related to previous injury to the knee | Damaged ligament; inflamed ligament; nerve type issue causing referred pain; | Pain signals danger; "I tend to take pain as a message to me from my body that something’s not right. So I try not to take painkillers... I feel like pain is my trigger to just try and do something a little bit different" **Q** |  |

**Theme 3: Coping with PHP**

|  | **Subtheme** | | | | | | | | | |  |  |  |
| --- | --- | --- | --- | --- | --- | --- | --- | --- | --- | --- | --- | --- | --- |
| **Participant** | **Economic** | **Calf stretches** | **Calf strengthening** | **Massage** | **Footwear** | **Orthoses/insoles** | **Taping** | **Modifying load** | **Injections/needling** | **Medications** | **Diet** | **Heat/ice** | **Other** |
| 1 |  |  |  | Self massage along the arch | No change with 'orthotic' work boots | Generic orthoses uncomfortable and pushed into the heel |  | Reduce activity when painful "generally speaking, when I feel the pain, I just stop doing that particular task" QQ |  |  |  |  | Socks |
| 2 |  |  |  |  |  |  |  |  |  | Panadol osteo ineffective |  |  |  |
| 3 |  | Calf stretching provides ST relief; | | Remedial massage; foam roller on the calf | Wear supportive shoes with built in arch support | orthoses | Taping provides short term relief but mechanism poorly understood | Rest from aggravating activities; work with a tolerable range; continue to maintain exercise. | PRP not effective |  |  | heat packs better than ice packs |  |
| 4 |  | calf stretches for ST relief - one minute hold 2-3 x day |  |  |  |  |  | Rest from aggravating activities "It’s just rest, I believe, that helped me a bit" **Q** |  | Voltaren provides short term relief |  |  |  |
| 5 | Costly socks, shoes, consultations | Calf stretches for ST relief |  |  |  |  |  |  |  |  |  |  |  |
| 6 |  |  |  | Self massage of the foot provides ST relief |  |  |  |  |  |  |  | Ice is uncomfortable |  |
| 7 |  |  | Strengthening has helped alleviate pain; 3 sets x 10-15 repetitions |  | Supportive shoes provide relief. *Go invest in a good pair of shoes firstly. Never walk barefoot or in thongs and even if you get up in the middle of the night, make sure you put a shoe on to walk around* ***QQ*** |  |  |  |  |  |  |  |  |
| 8 | Orthoses were expensive and ineffective; | |  |  |  | Generic orthoses expensive and ineffective and painful *"Well, talk about painful, they were dreadful and then most of my shoes, I couldn’t wear because my instep is too high and I couldn’t get the foot in the shoe with the orthotics in it"* **QQ** |  |  |  | Panadol provides ST relief |  |  |  |
| 9 |  |  |  |  | Footwear with some heel elevation "“So, when I get up in the mornings, I definitely put my shoes with the heel on, earlier than I would have normally” **Q** |  |  |  |  |  |  |  |  |
| 10 |  |  |  |  |  |  | Taping provided short term relief although associated with adverse reactions | Reduce the time standing to get pain relief |  | Topical NSAIDS ineffective |  |  |  |
| 11 |  |  |  |  | Supportive walking shoes effective | Generic insoles effective |  |  |  |  |  |  | Plantar fascia socks effective |
| 12 | Treatments are costly which encourages self diagnosis; loss of productivity | Calf stretching to alleviate pain in the short term; stretches on the stairs; | Calf strengthening 3 sets of 20 reps performed slowly | Spiky ball massage when its tight and painful; Chinese or Thai massage |  |  |  |  | Acupuncture provides short term relief |  |  | Rolling on a frozen styorfoam cup ineffective | Hot salty water socak sometimes effective |
| 13 |  |  |  | Self massage of the foot is effective | Footwear with some heel elevation |  |  | Reduced the amount of weightbearing exercise; avoid overstressing the foot |  |  |  |  |  |
| 14 |  |  |  |  |  |  |  | Started exercising (running) and stopped eating sugar which lead to weight loss and feeling more healthy "*“I didn’t realise that this would help but I’ve started running and I’ve stopped eating sugar and I don’t think the sugar has a relationship but what it has done is help me lose weight and get healthy*" **QQ** |  |  | Stopped eating sugar |  | Meditation; Reiki effective in the ST |
| 15 | Repeated consultations are expensive "*if you keep seeing them (clinicians) on a regular basis, it’s just so expensive. If I’d kept up the treatments with all of them for three – or two years, then struggle to pay the bills"* **QQ** | Calf stretching in the morning for 30 seconds |  | Self massage of the foot is effective | Avoid wearing flat shoes; supportive shoes (runners) |  |  |  |  |  |  |  |  |
| 16 | Orthoses were expensive and ineffective; state of the art runners were expensive and ineffective |  |  |  |  | Generic orthoses ineffective |  |  |  |  |  |  |  |
| 17 |  | Calf stretching in the morning; |  | Self massage of the foot in the morning |  | Interchange insoles in the shoes |  |  |  |  |  |  |  |
| 18 | Repeated consultations are expensive | Calf stretching ineffective in the ST | Calf stengthening ineffective in the ST *"Either I’m not particularly disciplined at doing that (stretching and strengthening) or it just didn’t actually help – maybe I was looking for a quick fix and it didn’t happen quickly enough and I became a bit frustrated with that*" **Q** |  | Shoes with small heels to keep the heel elevated | Generic orthoses effective *"the thing I’ve had the most success with is some orthotic inner soles that are very rigid and seem to hold my foot still. That seems to give me the most support*" **QQ** | taping effective in the ST but leads to skin irritation | On the role of rest *"maybe I just didn’t give it long enough, but I did have a week of total rest and it didn’t help my foot and my brain nearly went into a massive meltdown. So, no, rest just doesn’t work for m"* ***Q*** |  | Anti-inflammatories not effective in the ST |  |  | Dry needling was not effective |

**Theme 4: Sources of information**

|  | **Subtheme** | | | | |
| --- | --- | --- | --- | --- | --- |
| **Participant** | **Internet** | **Medical and allied health** | **Family and friends** | **People with shared experiences** | **Other** |
| 1 | Google; Richmond Football Club site | GP *I have to trust the information doctors give to me* ***Q*** |  | Can easily relate to people who have experienced the condition; issues described in layman's terms |  |
| 2 | Does not use the internet |  |  |  |  |
| 3 | Google; conflicting information *"Every now and then I get really, really frustrated and get back on and do another search and I find some different websites in that*". **Q** | GP; physiotherapist |  |  |  |
| 4 | Google - confusing content | GP "*Well, I just have to trust doctors. In this particular thing, I don't know anybody else who’s gone through it. So, I couldn’t rely on friends’ experience or anything like that. So, I couldn’t find out any more. So I just have to trust what the doctors tell me*" Q | Strong family support "I have a good family now that supports me a lot and understands my experience and knows what I’m going through. And that’s a big help" QQ | Can easily relate to people who have experienced the condition *"the idea is to gather as much information as possible from various sources regardless whether they are podiatrist, doctors, or friends, family, because sometimes you may get the best advice from people who – especially had experienced this sort of problem*" QQQ | Epsom salts, Magnesium tablets; krill oil |
| 5 | Minimal online searching - dubious of content | Osteopath |  |  |  |
| 6 | Google - Better Health Channel or myDr | Podiatrist; GP *"I did actually go to the doctor once and described it to him, but – yeah, he just said the massage as well. The GP didn’t look in the slightest bit interested, really"*. **Q** | Husband recommended massage |  |  |
| 7 | Google | Physiotherapist provided advice about exercises |  |  |  |
| 8 | Google |  |  | People with shared experiences |  |
| 9 | No online searching |  |  |  |  |
| 10 | Minimal online searching | Podiatrist and physiotherapist | Physiotherapist; podiatrist provided advice about orthoses and taping |  |  |
| 11 | No online searching | Podiatrist |  |  |  |
| 12 | No specific resource; internet to back up advice form others | Personal trainer "this guy is very big on mobility, flexibility,– and he’s just about going barefoot" Q |  | Internet to find information from people that have shared experiences |  |
| 13 | No online searching | Podiatrist; importance of face to face consultations *"Not everything that you read on the internet is possibly correct, so I just dealt with it. I mean, some physicians are not always correct too, but I just felt more secure in actually seeing someone and talking to them face to face rather than reading it off the internet"* **Q** |  |  |  |
| 14 | Google - resources limited |  | Friends with certain skills (Reiki) | neighbour with shared experience who recommended a visit to the GP |  |
| 15 | Google *"there was nothing really online to tell me how to deal with it"* ***QQ;*** Need more user friendly sites with simple terminology | Podiatrist, myotherapist, acupuncturist; Podiatrist who provided advice about footwear; |  |  |  |
| 16 | No online searching |  |  |  |  |
| 17 | No online searching. *A lot of description of very long words that I didn’t really understand and I – so, it gave a description about what it was, but actually I didn’t find anything was really telling me what to do* ***QQ*** | Podiatrist; GP |  |  |  |
| 18 |  | Podiatrist; physiotherapist; |  |  | Anyone with advice |

**Theme 5: Patient needs**

|  | **Subtheme** | | |
| --- | --- | --- | --- |
| **Participant** | **Delivery mode for learning** | **Education** | **Pain relief** |
| 1 | Face to face and online; "*Well, in this case, only if I saw someone one on one like yourself someone who’s a qualified podiatrist or somebody else, then you can have a chat about it, but I think online is perfectly fine if – yeah, if it can help people"* ***Q****.* | List of exercises to perform; | Methods to stop the pain "*there may be no magic bullet here to say we can get rid of it, but if there's a way to stop the pain, so you can be more active"* ***Q*** |
| 2 | Face to face. *"A lot of people use the internet these days. I prefer speaking to somebody about it*". **Q** | Understand the cause; treatment options |  |
| 3 | Face to face supplemented with online | Education regarding a graded exposure to exercise; explanation of the cause and underlying pathology *"If I’ve had a better idea, better understanding of what was actually happening with it, I think that would have guided me a lot better in what I was doing about it"*. **Q** |  |
| 4 | Face to face supplemented with some online; "But having an area where you can go there – is simple, because again, as I mentioned to your earlier, that – well, there are obviously a lot of older people that are experiencing this. So, most of them don’t have the technology". Q | Clear explanation of the pathology to inform treatment decisions; treatment options; long term solutions; discussion of prognosis; what works, what doesn't work. |  |
| 5 | Face to face with handouts *"I want some clear information. Maybe I’ve seen in some instances for other situations where people have a hand-out and there’re a couple of little photos with brief explanations and maybe the five top suggestions, something to take home. They probably explained it to you at the time but some of them I haven’t seen for months, so something concrete to walk out of there that you can check and refer to later on"*. **QQ "***I haven’t really gone too much to the internet because I’m very dubious of what’s uploaded online. I don’t know a particular site or an organisation that I would think would have great, quality information, but I would like to know about it if it exist"* ***QQ "****My advice would be maybe don’t go online. I know that’s probably the first thing that people do but I’d be looking firstly at footwear and stretching and then muscle strength, yeah". QQ* | Clear explanations; | Adress the underlying issue rather than focus too much on proximal issues that might be associated with the condition |
| 6 | Online; handouts *"I suppose, but written pamphlets would be good or reference to a – I don't know – a website or something like that that can tell me what I'm meant to be doing".****Q*** | Information sheets; links to useful resources | To eliminate the pain |
| 7 | face to face |  | Speed up the process |
| 8 | Face to face and internet | Match treatments to beliefs and expectations of patients; understand the cause; prognosis; appropriate questioning to elicit information that identifies the underlying cause |  |
| 9 | No types of formal learning | Treatment plan; |  |
| 10 | face to face supplemented with online | Education about causal factors; underlying problem; prognosis *"I know that’s a very subjective sort of thing, but to have some idea of – okay, this could take months, it could take three months, it could take six months – just to have some sort of idea, I think, of how long it might take ‘cause I really thought I’d be over it by now"* **Q** |  |
| 11 | Face to face; does not use the internet | Treatment options; | To cure the heel pain |
| 12 | Face to face and online *"Yes, so it’s been – so when I have these conversations then of course the place to go is the internet, and the source of data, and then that then, I guess, backs up or not either way, the – I guess, the information I get fed from people" Q* | Explanation of the cause, pathology; imaging to view the underlying issues *"Unless you actually see inside and what it is, how do you know that’s the real issue for me*? **Q**; correct diagnosis; treatment options |  |
| 13 | *Not everything that you read on the internet is possibly correct, so I just dealt with it. I mean, some physicians are not always correct too, but I just felt more secure in actually seeing someone and talking to them face to face rather than reading it off the internet"* **Q** | Simple explanations; firm, confident, diagnosis; encourage active patient involvement; succinct recommendations |  |
| 14 |  | Treatment options prior to considering surgery; options with different levels of evidence |  |
| 15 | Face to face and online | Education regarding causal factors and prognosis; | Eliminate the pain |
| 16 | Face to face no online | Clear explanations |  |
| 17 | Face to face | Clinicians to solve the problem in addition to providing pain relief; provide treatment options including exercises | Eliminate the pain and get resume normal function |
| 18 | Face to face and online *"The physio delivered a little bit. He’s a physio that deals with a football club as well and so I figure when he’s seeing it in – he’d be seeing this sort of thing a lot with footballers that – ‘cause why wouldn’t I heal like a footballer? Why wouldn’t it be relevant for me? And I also had education from my podiatrist and I would say that there was education from online, but I’m not sure how trustworthy some of that is*. | A developed plan; long term plan; understand the prognosis; treatment options; a treatment plan that is tailored to the individual; appreciating the needs and beliefs of the patient; |  |

**Theme 6: Advice to others**

|  | **Subtheme** | | | | |
| --- | --- | --- | --- | --- | --- |
| **Participant** | **Seek advice** | **Footwear** | **Load management** | **Treatment** | **Other** |
| 1 | Get seen straight away |  | Don't continue to run on it |  | Get the heel scanned |
| 2 | See a doctor |  |  |  |  |
| 3 | See a specialist; get an accurate diagnosis *"Get a good diagnosis and someone who knows what they're doing. I suppose looking back, getting a firmer diagnosis at the beginning maybe, trying to get some information that seemed to relate more specifically to your own condition because I found that I was never really quite clear on what advice I was getting really related to what I had because of confusion over what it was*" **Q** |  |  |  | Regrets losing so much conditioning; dealying a definitive diagnosis |
| 4 | Seek advice from GP, podiatrist, friends, family, people with shared experiences |  |  |  |  |
| 5 |  |  |  |  | Don't go online; consulting with the osteopath over such a long period of time with no relief; not doing the exercises; not addressing the source of the problem |
| 6 |  |  |  |  |  |
| 7 | Strengthen the calf muscles | Avoid barefeet; wear supportive shoes; | |  |  |
| 8 |  |  |  |  | Regrets purchasing orthoses |
| 9 |  |  | Don't push through the pain |  | Would not presume to have enough knowledge to provide advice |
| 10 | Seek advice from more than one clinician |  |  | A process of trial and error | No regrets - just part of the experience |
| 11 | Seek advice from the GP; get some imaging; | Wear supportive shoes |  |  | Get an ultrasound |
| 12 | See a specialist; educate yourself |  |  |  | Regrets not seeing a specialist earlier |
| 13 | See a podiatrist "*the longer you leave it, the worse things become*" **Q** |  |  |  |  |
| 14 | See a specialist |  | Exercise to keep moving and lose weight |  | Regrets wearing poor footwear in teenage years |
| 15 | See a specialist; podiatrist or myotherapist |  |  |  | No regrets - all interventions are worth a try |
| 16 |  |  |  |  |  |
| 17 | See a specialist; get some imaging |  |  |  |  |
| 18 | Be patient; find a good practitioner; |  |  |  | Regrets walking barefoot; not getting orthoses earlier |

**Theme 7: Patient unmet needs**

| **Participant** | **SUBTHEME (CODE)** | | | |
| --- | --- | --- | --- | --- |
|  | **Guidance** | **Reliable sources** | **knowledge of evidence base** | **Other** |
| 1 | Uncertainty about nature of the cause; no sources that provide definitive options | Uncertain who to see or sites to visit; sites not trustworthy; | Uncertain; Corticosteroid effective; online information is not definitive; | GPs need to be more educated "I believe maybe if the GPs are more educated about – yes, there are options there – and maybe them actually referring to a podiatrist or someone specialised would be great" **Q** |
| 2 | Better understanding of cause; treatment; prevention. |  | Uncertain *"I know nothing, absolutely nothing about any of it"* **Q** |  |
| 3 | Improved explanation of treatments; Better explanation of the pathology *"a lot of the education is being well, “Do this or do that,” but without really explaining what it is that you’re doing and why you’re doing it and what you're supposed to achieve"* **QQ** Conflicting information *"I mean there’s so much conflicting information on the internet, trying to put that into context with what doctors told me, what my physio friend at work told me and reading different things on the internet, trying to relate that to my condition, and work it all out”***. QQ** |  |  |  |
| 4 | Better explanation of the underlying pathology; typical signs and symptoms |  | Limited knowledge; |  |
| 5 | Better explanation of treatments and why they should be performed; lack of treatment direction; list of effective treatment options *"Here’s half a dozen things that you should try in order of how frequently they assist people with similar problems,” I think. So like a bit of a format or a plan of what to do and when to move to the next step."* ***QQQ*** |  | Calf stretching and strengthening mean to be effective |  |
| 6 |  |  | Limited knowledge; massage meant to be effective; |  |
| 7 | What to do; treatment options; |  | No knowledge of the evidence; |  |
| 8 |  |  |  |  |
| 9 |  |  | No knowledge |  |
| 10 | Confusion about the balance between exercise and rest as part of the treatment program | | Corticosteroid injections meant to be effective |  |
| 11 |  |  |  |  |
| 12 | Better explanation of cause, pathology ; who are the specialists in this area? Need to get to the root cause of the issue; differences in the UK and AUS healthcare system. In the UK, the GP acts as the referrer. In Aus, everyone is a doctor - uncertainty about who to see in Aus | Need reliable sources of information from an Australian perspective | Uncertain; orthoses; surgery if you have a heel spur; |  |
| 13 |  |  | Dry needling; foot orthoses and corticosteroid injections are meant to be effective |  |
| 14 | Better understanding of the pathology; treatment options; | No online reliable sources evident; online resources with simple terminology | Limited knowledge; online information weighted toward surgery |  |
| 15 | Education limited to footwear advice and massage; guidance around cause, prognosis, treatment plans; what to do when not responding to treatment *"Look, this could be something that is long-term. This could be something that you might need to – have to deal with for a couple of years or -” I never got that impression that it was something that could go on for a while. I think – after, I think, the third or fourth time, she didn’t even make another appointment for me so I thought “Was that it? Is that it now? So what do I do now?”* **QQ** | Need more user friendly sites with simple terminology | Uncertain; massage and insoles meant to be effective. |  |
| 16 |  |  | Corticosteroid injections meant to be effective; foot taping |  |
| 17 |  |  |  |  |
| 18 | Defining who are the specialists; consistency in treatment approach; matching the treatment plan to the stage of injury; providing ST, MT and LT options. Information about management is conflicting and uncertain *"But honestly, if someone told me to mix up a special drink ‘cause that’s what was gonna fix it, that’s probably what I’d do as well. So, I probably am trying anything. It’s a bit like spin the wheel and try your luck. I’ll try it all"* **QQ**; everyone has an opinion "*Some people say rest, some people say keep moving, some say stretch, some say don’t stretch"* **Q**; prognosis is unacceptable; logistics of taping not explained; | Online material is complicated *"a lot of description of very long words that I didn’t really understand and I – so, it gave a description about what it was, but actually I didn’t find anything was really telling me what to do"* **QQ'** | Calf stretching and strengthening should be effective; rest |  |

**Theme 8: Interest in online education**

|  | **Subthemes** | | | |
| --- | --- | --- | --- | --- |
| **Participant** | **Online features** | **Online content** | **Reputable** | **Other** |
| 1 | Engaging with audio and visual input | Exercise treatment plans; diagnostic features to ensure accurate self diagnosis *"Yeah, that's my pain,” or, “That’s what I've got.”* ***Q*** | The site needs to be reputable; university alignment |  |
| 2 | Don't use the internet | Causal factors; how to fix it. |  |  |
| 3 | Depth of knowledge *“I know some people are only looking for something very shallow and overall they don’t want to be bamboozled by too much, but I’m one of those people I want to go down through the levels, and it’s not telling me enough. I want to know more and more detail”* ***QQQ;*** *Pretty much all of the websites that I looked at had the same sort of material just in different words, different diagrams, but not really getting down any deeper than that, and I think I really wanted to understand a bit more about it* ***QQQ*** |  |  |  |
| 4 | Diagrams; written text; no videos to demonstrate exercises; information fact sheets to download | Definition of the condition and the classic presentation to help diagnosis; strategies to alleviate pain |  |  |
| 5 | Diagrams; simple terminology; based on the best evidence | Definition of the condition and the classic presentation to help diagnosis |  |  |
| 6 | Videos of exercises; | Definition of the condition; treatment options; education about modifying load on the heel - rest versus exercise; footwear advice |  |  |
| 7 | Videos; diagrams | Demonstration of exercises; diagnostic features; footwear advice |  |  |
| 8 | Audio; an audio walk through the site | An introduction to heel pain with a succinct summary; cause of PHP; questions to ask to help with a self diagnosis; treatment options; what to do; treatment direction during different stages | Aligned to a reputable university |  |
| 9 | Videos; instructions for exercises; | Encouragement that the condition will improve | Designed by experts; from a reputable organisation; |  |
| 10 | Videos, text, information fact sheets that can be downloaded | Definition of the condition; treatment options; education about modifying load on the heel - rest versus exercise; footwear advice |  |  |
| 11 | Doesn't use the internet | Information regarding supportive shoes; insoles for shoes; |  |  |
| 12 | Diagrams; patient stories with shared experiences | images that demonstrate the classic presentation; what specialists to see "I think that that’s been the key for me is really knowing who to go and see" **Q**; defining the specialists; treatment options; |  |  |
| 13 | Diagrams | Images that demonstrate the classic presentation; exercise instructions; what to do; what not to do; causes of heel pain | A reliable, accredited resource *"You’d probably want to know if it’s accredited, so if it’s coming from a reliable source"* **Q** |  |
| 14 | Simple terminology | Diagnostic features |  | Easy to access and free |
| 15 | Audiovisual; diagrams; text | Treatment options; cause of PHP |  |  |
| 16 | Audiovisual; diagrams; outcomes of different treatments; handouts that could be downloaded | Treatment options |  |  |
| 17 | Hard copies/handouts that coud be downloaded | Defining the specialists; treatment options; exercise options |  |  |
| 18 | Videos to demonstrate treatments; access to more detailed information; succinct descriptions; links to relevant associations; content produced by an expert. *I want videos that show where pain is on the foot, having that pointed out – drawn on a foot, so I can replicate it on mine, all that sort of – if your pain is here, it could be this. If it’s here, it’s probably not this* ***QQQ*** | Treatment plans in the ST, MT and LT; what to do; what not to do in regards to treatment | A site linked to a podiatry association, hospital, or a facility that has expertise in the area | Easy to access |
